# Atypical Presentation of Subarachnoid Hemorrhage (SAH): A Case Report

**DOI:** 10.7759/cureus.103266

**Published:** 2026-02-09

**Authors:** Nader Abdelrazig, Muhammad Khizar Hayat, Shehzad Safdar, John Caird

**Affiliations:** 1 Neurosurgery, Beaumont Hospital, Dublin, IRL; 2 General and Colorectal Surgery, Beaumont Hospital, Dublin, IRL; 3 Neurosurgery, Punjab Institute of Neurosciences, Lahore, PAK

**Keywords:** aneurysm, atypical presentation, delayed diagnosis, elderly, endovascular coiling, subarachnoid hemorrhage

## Abstract

Subarachnoid hemorrhage (SAH) is a life-threatening condition, often caused by an aneurysmal rupture. Generally, it presents with classical symptoms of thunderclap headache, along with other neurological findings; however, atypical presentations can also occur, hence leading to a delay in diagnosis, particularly in elderly patients.

We present a case of a 71-year-old woman diagnosed with acute pancreatitis. Although her gastrointestinal symptoms improved, her confusion persisted, which prompted further investigation, leading to the diagnosis of SAH. This case report highlights the atypical presentation of SAH in the elderly population and urges clinicians to keep a high index of suspicion for such occurrences.

## Introduction

Subarachnoid hemorrhage (SAH), which involves bleeding into the subarachnoid space, is a life-threatening neurological emergency, the most common cause of which is a ruptured intracranial aneurysm. Although it represents a minority (5-10 percent) of all stroke cases, SAH is over-represented with regard to mortality and long-term disability caused by stroke. Global epidemiological statistics show that the incidence of aneurysmal SAH is 6 to 10 cases per 100,000 person-years, with substantial disparities between regions and populations [1]. It occurs predominantly in women, with a female-to-male ratio of approximately 3:2, and in persons aged between 40 and 60 years [2]. Although treatment procedures have improved, outcomes remain poor; mortality is reported to range between 30 percent and 50 percent, and approximately 50 percent of survivors report permanent neurological or cognitive disability [3].

The typical signs of aneurysmal SAH are an abrupt, intense form of the so-called thunderclap headache, along with nausea, vomiting, photophobia, neck stiffness, and even unconsciousness. Conversely, elderly patients usually have non-specific and atypical symptoms that may cloud the diagnosis. Most symptoms in this demographic can take the form of confusion, disorientation, abdominal pain, or an overall feeling of malaise, which does not always have a localized neurological correlate [4]. Such an unclear clinical picture increases the risk of delayed or incorrect diagnosis.

Non-contrast computer tomography (CT) of the brain is the initial imaging test performed in people suspected of having SAH. Its sensitivity is greatest in the first six hours after the onset of symptoms, approaching 98-100 percent [5]. Thereafter, as sensitivity decreases, a negative CT scan in the context of ongoing clinical suspicion will necessitate a lumbar puncture (LP). This test must be carried out a minimum of 12 hours following symptom onset to reliably identify the presence of xanthochromia or a persistent rise in the number of red blood cells per sample tube [6].

After a diagnosis of SAH, vascular imaging must be conducted to determine the origin of bleeding. Computed tomography angiography (CTA) is a rapid and non-invasive test. Nonetheless, the gold standard remains digital subtraction angiography (DSA), which has better resolution and is capable of simultaneous endovascular or surgical treatment (i.e., aneurysm coiling or clipping) [7]. Confirmed aneurysmal SAH requires admission to a neurocritical care unit, where multidisciplinary monitoring is required to reduce secondary insults.

The case report describes the clinical course of a 71-year-old female in whom the diagnosis of SAH was delayed because of an initial presentation with predominantly gastrointestinal symptoms and disorientation. The case highlights the importance of using a diagnostic framework based on systematic assessment of elderly patients with non-specific symptoms, as well as the need to maintain a high index of suspicion to ensure that this serious condition is diagnosed at an early stage.

## Case presentation

**Figure 1:**
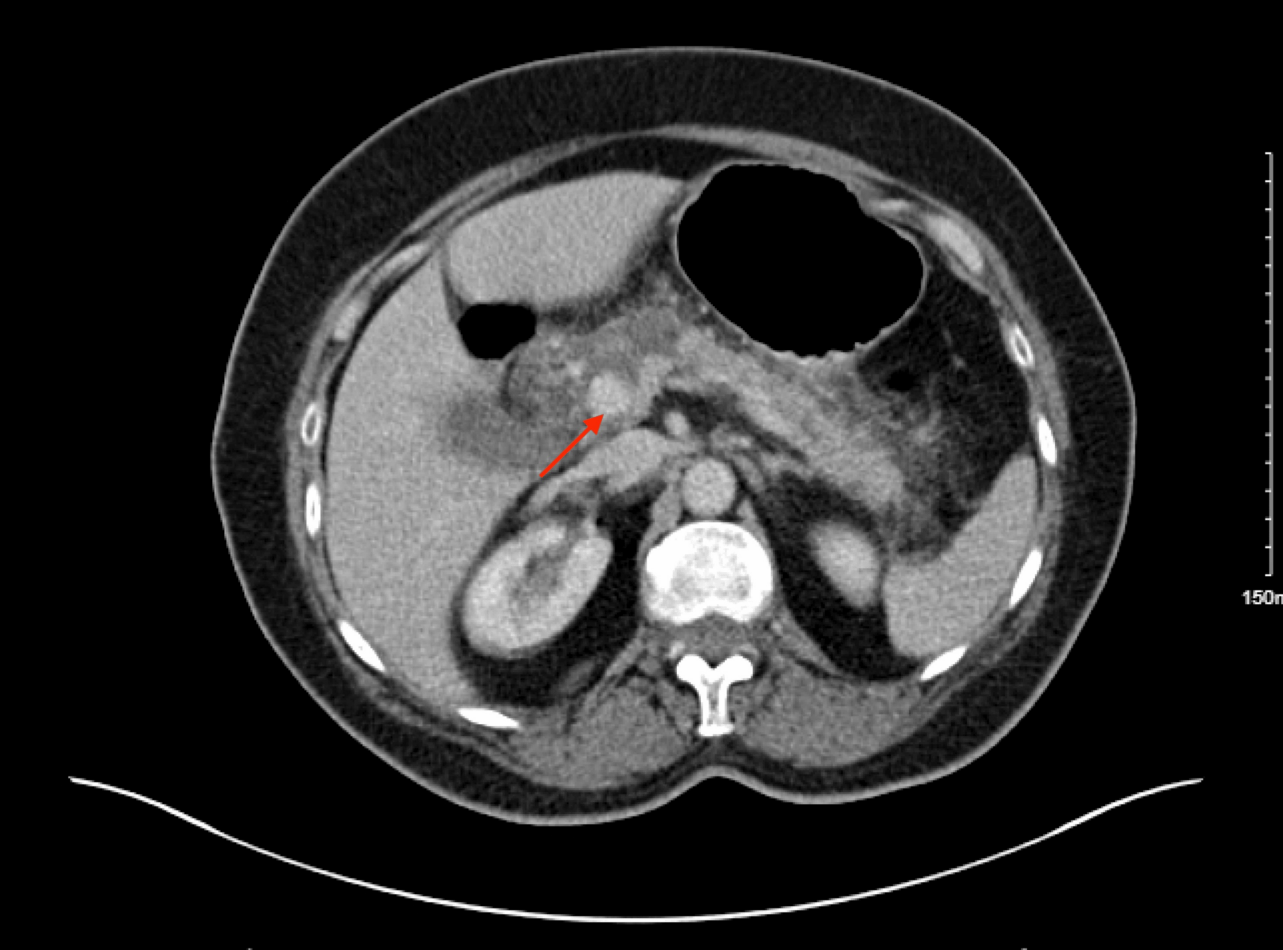
CT abdomen and pelvis showing fat stranding consistent with acute pancreatitis (red arrow). CT, computed tomography

A 71-year-old female patient with a documented medical history of hypertension, hypercholesterolemia, and hypothyroidism was admitted with a primary complaint of abdominal pain ongoing for two days. Initial evaluation, including an abdominal CT scan (Figure 1) and elevated serum amylase levels (Table 1), performed as part of the Emergency Department assessment, led to a diagnosis of acute pancreatitis. She was managed conservatively with supportive care, including intravenous fluids and analgesia, and her blood tests were monitored for progress. While her abdominal symptoms and laboratory markers subsequently improved, the patient exhibited persistent confusion despite supportive treatment, leading to the decision to perform a CT brain scan.

**Table 1:** Bloods at initial presentation. LDH, lactate dehydrogenase

Lab Test	Result	Reference Unit
Hemoglobin	13.6 g/dL	12 - 15 g/dL
White Blood Cells (WBC)	9.3 x 10^9^/L	4 - 10 x 10^9^/L
Platelets	249 x 10^9^/L	150 - 410 x 10^9^/L
C-Reactive Protein	1.1 mg/L	0 - 5 mg/L
Amylase	120 m/L	28 - 100 m/L
LDH	269 m/L	0 - 247 m/L
Calcium	2.46 mmol/L	2.20 - 2.65 mmol/L

**Figure 2:**
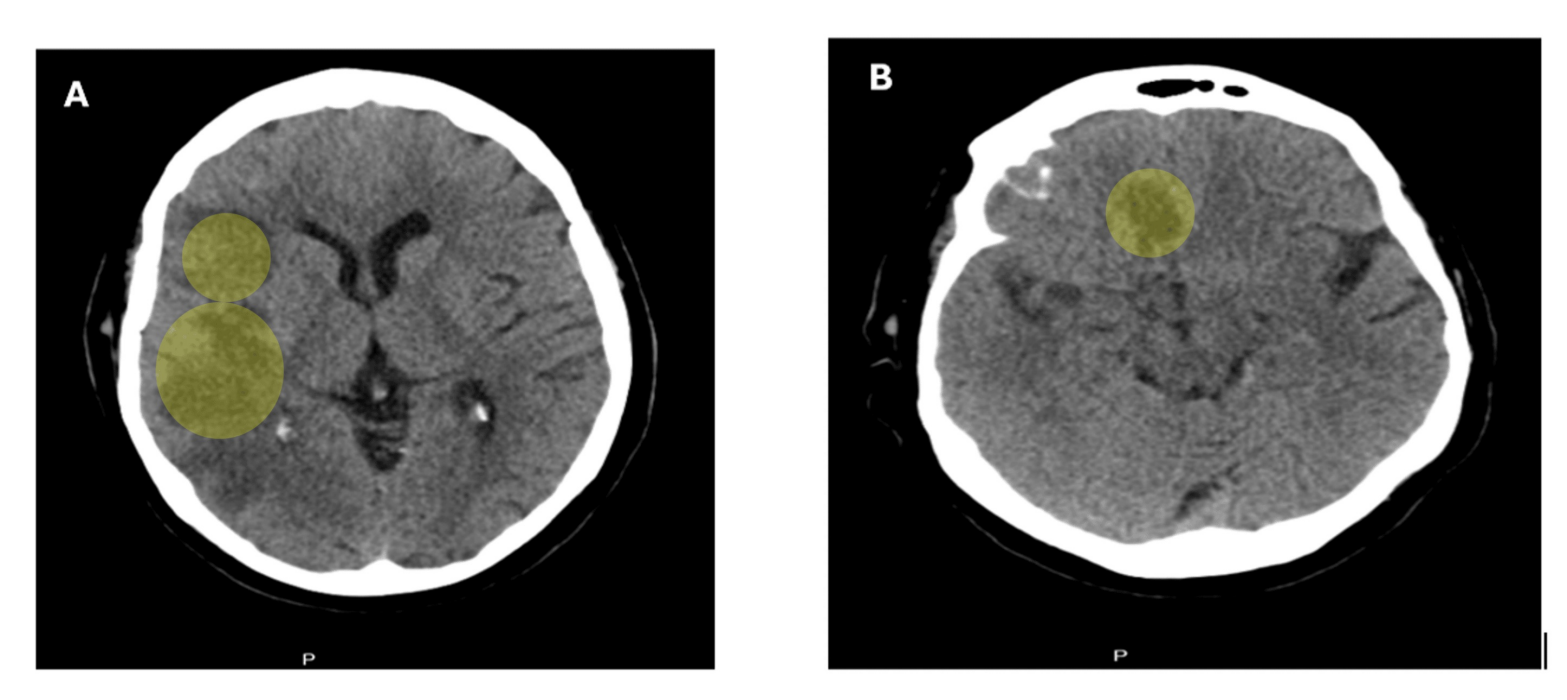
(A) Hypodensities in the right MCA territory involving the frontal, temporal, and parietal lobes on non-contrast axial CT of the brain (yellow highlight). (B) Hypodensity in the left frontal parafalcine region on non-contrast axial CT of the brain (yellow highlight). MCA, middle cerebral artery; CT, computed tomography

Neurological examination was performed, which revealed no focal deficits. The non-contrast CT scan of the brain (Figures 2A-2B), performed seven days after symptom onset, demonstrated hypodensities in the left frontal parafalcine region and the right middle cerebral artery (MCA) territory. 

**Figure 3:**
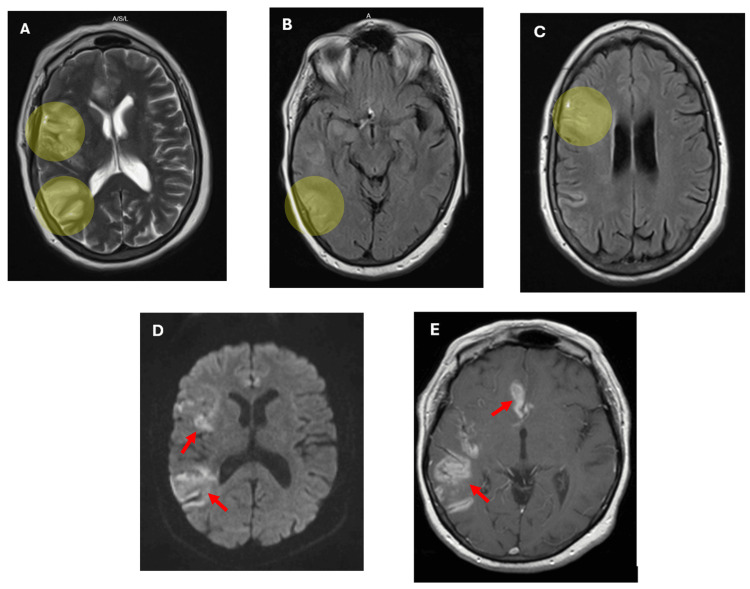
(A) Hyperintensity in the right MCA territory on axial T2-weighted MRI of the brain (yellow highlight). (B) FLAIR hyperintensity in the right temporal lobe on axial FLAIR sequence MRI of the brain (yellow highlight). (C) FLAIR hyperintensity in the right frontal lobe on axial FLAIR sequence MRI of the brain (yellow highlight). (D) Restricted diffusion in the right MCA and ACA territories, involving the right frontal and parietal lobes and the frontal parafalcine area, on DWI B1000 sequence axial MRI of the brain (red arrows). (E) Areas of enhancement in the right frontal and parietal lobes on axial T1-weighted post-contrast MRI of the brain (red arrows). MCA, middle cerebral artery; ACA, anterior cerebral artery; MRI, magnetic resonance imaging; FLAIR, fluid-attenuated inversion recovery; DWI, diffusion-weighted imaging

Magnetic resonance imaging (MRI) of the brain (Figures 3A-3E) was performed the following day and demonstrated restricted diffusion in the right MCA and anterior cerebral artery (ACA) territories, with sparing of the right basal ganglia. Additionally, vasogenic edema and fluid-attenuated inversion recovery (FLAIR) hyperintensity were observed near the anterior horn of the right lateral ventricle. A small volume of SAH was also detected in the right frontal lobe and interhemispheric fissure. The differential diagnosis included ischemic stroke, atypical infection, vasculitis, and neoplastic processes.

Due to the presence of a small-volume SAH and a wide range of differentials, an LP was performed to exclude infectious and autoimmune processes as well as SAH. The results were negative (Table 2).

**Table 2:** Lumbar puncture CSF results. CSF, cerebrospinal fluid; RBC, red blood cells; WBC, white blood cells; Sp, specimen

Lab Test	Result	Reference Unit
CSF Protein	0.46 g/L	0.15 - 0.45 g/L
CSF Glucose	3.6 mmol/L	2.2 - 4.4 mmol/L
CSF Viral Screen	Negative	Negative/Positive
CSF Culture	No organisms seen	-
CSF Microscopy Sp 1	WBC = 22/cmm, RBC = 126/cmm	WBC <5/cmm, RBC = 0/cmm
CSF Microscopy Sp 2	WBC = 14/cmm, RBC = 7/cmm	WBC <5/cmm, RBC = 0/cmm
CSF Microscopy Sp 3	WBC = 33/cmm, RBC = 17/cmm	WBC <5/cmm, RBC = 0/cmm

**Figure 4:**
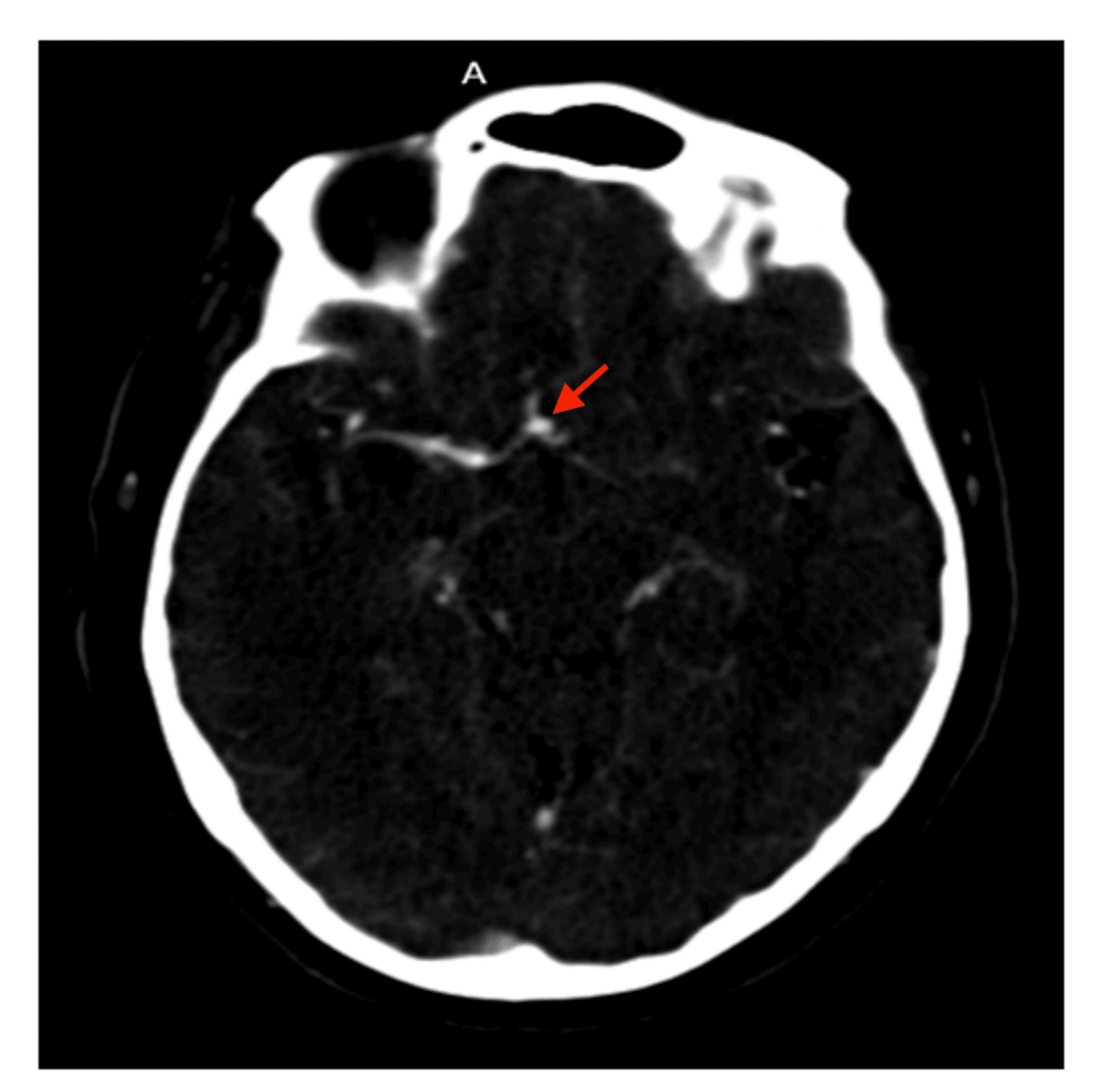
Dilatation of the proximal segment of the ACA, measuring 4 mm and suggestive of a suspected aneurysm in the right ACA or at the ACOM, along with irregularity in the right MCA, as seen on axial arterial-phase CTA of the brain (red arrow). ACA, anterior cerebral artery; ACOM, anterior communicating artery; MCA, middle cerebral artery; CTA, computed tomography angiography

Following the LP results, the case was discussed with the neurosurgery team to consider a brain biopsy. The advice was to perform an urgent CTA of the brain before consideration for biopsy. She subsequently underwent a CTA of the brain (Figure 4) due to the MRI findings, which revealed a suspicious 4 mm aneurysm in the right ACA and irregularity in the right MCA, with no evidence of vascular occlusion.

Her case was also discussed with the neurointerventional radiologist. It was advised to proceed with DSA to exclude a delayed aneurysmal SAH in the presence of an anterior communicating artery (ACOM) aneurysm, with resultant vasospasm and infarction. A retrospective history review revealed that the patient had experienced a sudden headache and abdominal pain prior to her initial assessment, as she was confused at the time of evaluation.

**Figure 5:**
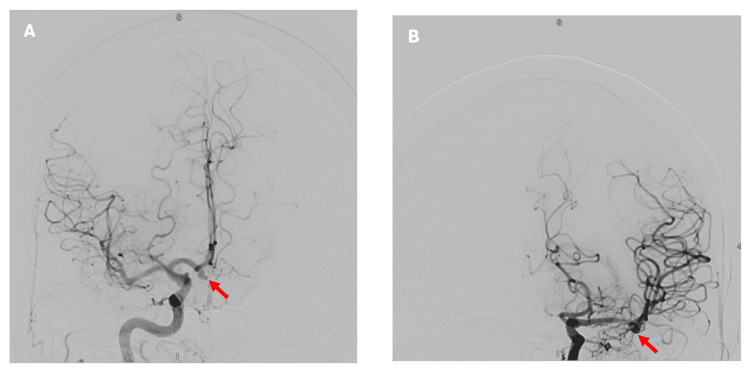
(A) A 4 × 3.5 × 3.5 mm aneurysm arising from the junction of the right A2 segment and the ACOM, with a neck measuring 3 mm, seen on DSA following right ICA injection (coronal view - red arrow). (B) An incidental aneurysm of the left MCA, measuring 2 × 2.7 × 2.5 mm, seen on DSA following left ICA injection (coronal view - red arrow). ACOM, anterior communicating artery; DSA, digital subtraction angiography; MCA, middle cerebral artery; ICA, internal carotid artery

**Figure 6:**
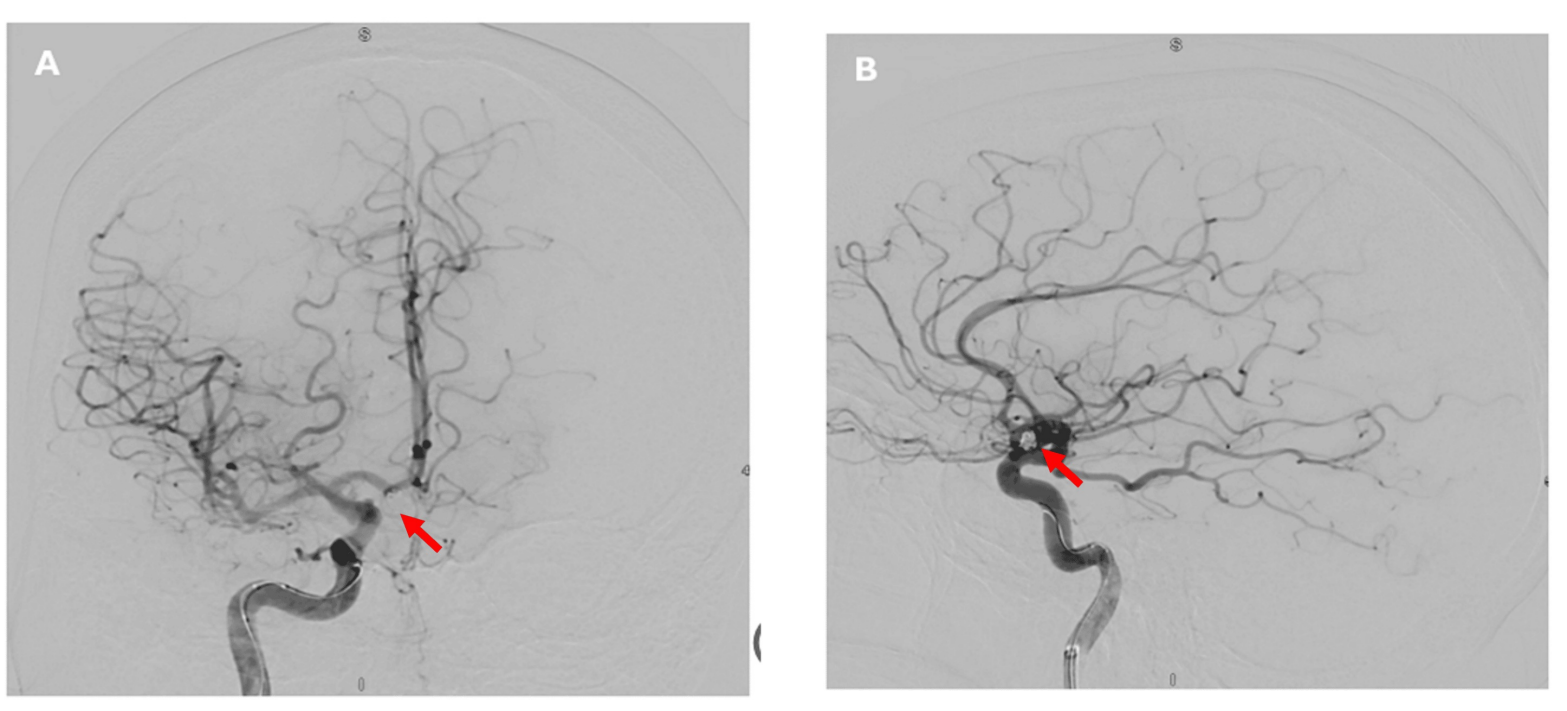
(A) Successful endovascular coiling of the ACOM aneurysm, seen on DSA following left ICA injection (coronal view - red arrow). (B) Lateral view of DSA following left ICA injection, demonstrating the post-coiling appearance of the ACOM aneurysm (red arrow). ACOM, anterior communicating artery; DSA, digital subtraction angiography; ICA, internal carotid artery

DSA confirmed the presence of a 4 mm ACOM aneurysm and identified an incidental 3 mm aneurysm at the left MCA bifurcation (Figures 5A-5B). The ruptured aneurysm was successfully treated with endovascular coiling (Figures 6A-6B).

**Figure 7:**
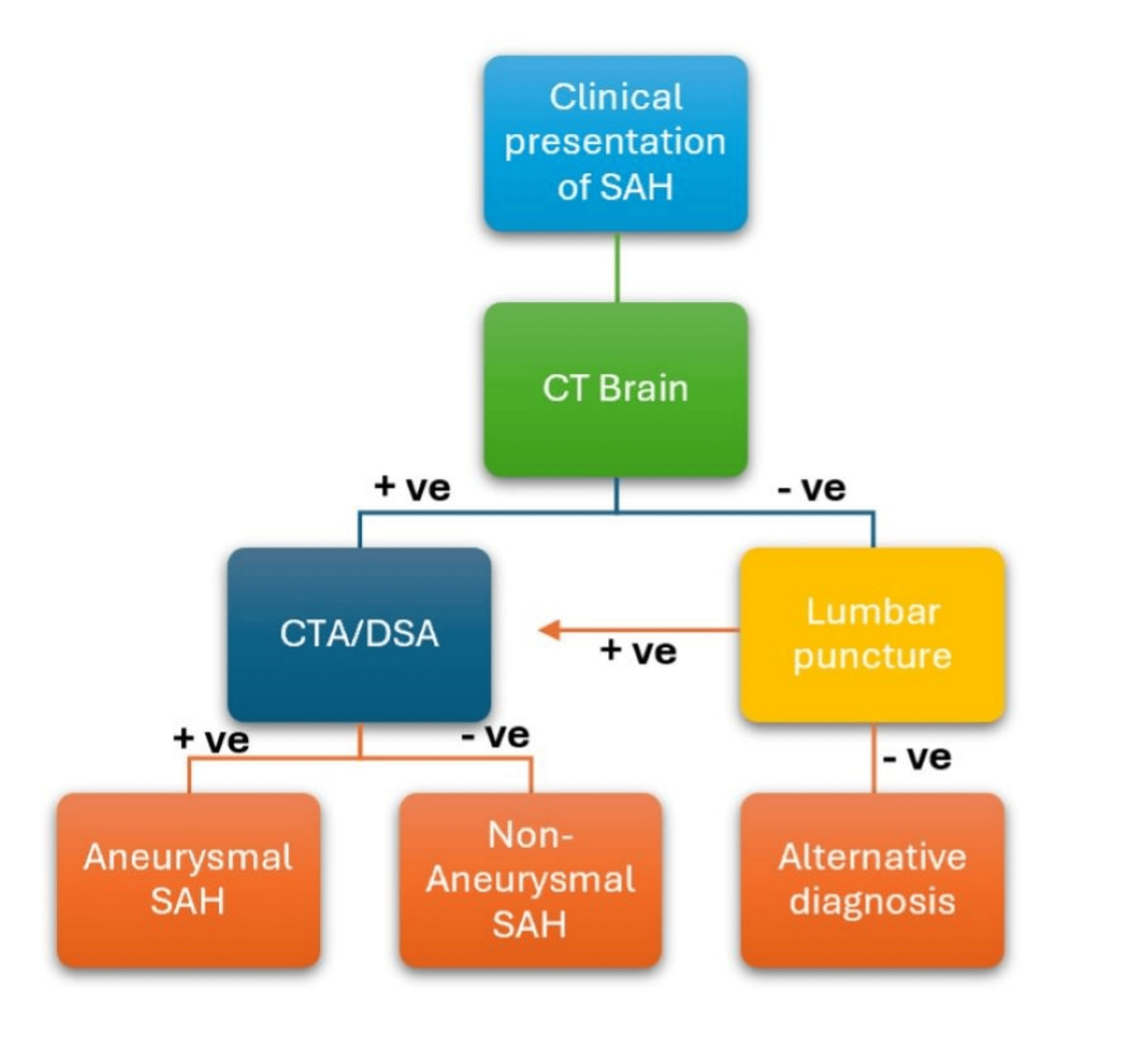
A structured algorithm combining clinical examination and neuroimaging.

To address this diagnostic complexity, it is essential to employ a structured algorithm that combines clinical examination and neuroimaging to manage the situation in a timely manner (Figure 7).

Outcome and follow-up

Post-intervention, the patient remained neurologically intact, with a Glasgow Coma Scale (GCS) score of 15 and no focal neurological deficits [8]. However, cognitive impairment persisted, particularly affecting short-term memory. The case was reviewed at the neurovascular multidisciplinary team meeting, and it was recommended that she undergo follow-up with magnetic resonance angiography (MRA) of the brain in six months. She was subsequently transferred to her local hospital for cognitive rehabilitation.

## Discussion

To prevent life-threatening complications and irreversible brain injury, prompt diagnosis and management of SAH are essential. It presents with varied symptomatology, with a classical presentation of sudden-onset, severe headache, often described as a thunderclap headache. Vomiting, photophobia, and loss of consciousness are other symptoms that commonly accompany the headache. In the elderly population, these symptoms may not be present, and the patient can present with atypical features, hence increasing the risk of diagnostic delay.

In this case, factors that led to delayed diagnosis include, firstly, age-related physiological changes and comorbidities of the patient. Age-related physiological changes include cerebral atrophy, which increases intracranial compliance, hence blunting the sudden rise in intracranial pressure (ICP). This sudden rise in ICP is responsible for the thunderclap headache, which may be absent in elderly patients [9]. Another explanation is the decline in neuronal sensitivity, which significantly decreases due to neurodegeneration and demyelination, hence increasing tolerance to irritation [10].

Comorbidities can mask or alter the typical clinical picture. Confusion, lethargy, and abdominal pain are other non-specific symptoms that can present in the elderly population, as opposed to the classical thunderclap headache [11]. Secondly, as this patient did not have any focal neurological deficits, an alternative diagnosis was more likely, especially given the presentation with abdominal pain. This led to an initial diagnosis of acute pancreatitis, delaying further neurological assessment. Lastly, due to the initial improvement or partial symptom resolution, particularly with supportive care, a false sense of clinical stability and reduced suspicion for central nervous system pathology developed. A study published by Wen et al. looked at atypical presentations of SAH and compared them with typical presentations. Abdominal pain was not among their review of 86 patients with atypical presentations in individuals over 70 years of age [9]. This case report highlights another important symptom to increase suspicion of delayed SAH.

Similar challenges have been described in the literature. Goyal et al. reported a patient with SAH secondary to rupture of an ACOM aneurysm, who had abdominal distension and sepsis, and was found to have acute pancreatitis based on CT abdomen findings and a high serum lipase level, despite a normal serum amylase level, which was likely triggered secondary to systemic inflammatory response syndrome (SIRS) [12].

Stress-related mechanisms could be the reason for SAH, potentially leading to acute pancreatitis. As SAH is associated with triggering a systemic inflammatory response and a catecholamine surge, this could have indirectly contributed to pancreatitis. Widespread endothelial injury, vascular impairment, and splanchnic vasoconstriction are predisposing factors for abdominal organ ischemia. Reduced organ perfusion, along with increasing pro-inflammatory cytokines, can lead to pancreatic injury [13,14].

Although a direct causal relationship cannot be definitively established, several factors support pancreatitis occurring secondary to SAH in this case. The common etiologies of acute pancreatitis, such as gallstone disease, alcohol use, metabolic disease, medication exposure, and infection, were excluded [15]. The temporal association between the onset of neurological symptoms, the development of SIRS and catecholamine surge, and subsequent pancreatitis supports a stress-related mechanism secondary to SAH [16].

To avoid missed or delayed diagnoses, several key strategies should be implemented in clinical practice. These include maintaining a high index of suspicion for SAH in elderly patients presenting with atypical symptoms, despite the absence of a classical thunderclap headache or any focal neurology. Early neuroimaging, particularly a non-contrast CT of the brain, should be performed in cases of altered mental status without an evident cause. Even if the CT is non-diagnostic but clinical suspicion remains high, an LP should ideally be performed at least 12 hours after symptom onset to evaluate for xanthochromia. A definitive management plan should be put in place early once hemorrhage is confirmed, with CTA or DSA used to identify the source, and a multidisciplinary approach can also be adopted in diagnostically challenging cases [17].

## Conclusions

Minimizing diagnostic delays is critical for favorable neurosurgical outcomes post-SAH if appropriately managed. Although a delayed diagnosis can still result in adequate recovery, early recognition and prompt intervention can help reduce the risk of long-term cognitive sequelae and improve overall functional prognosis. A comprehensive approach should be taken toward patient diagnosis.
